# Results from a Swedish model-based analysis of the cost-effectiveness of AI-assisted digital mammography

**DOI:** 10.1007/s00330-025-11821-9

**Published:** 2025-07-19

**Authors:** Johan Lyth, Pantelis Gialias, Magnus Husberg, Lars Bernfort, Tomas Bjerner, Maria Kristoffersen Wiberg, Lars-Åke Levin, Håkan Gustafsson

**Affiliations:** 1 Department of Health, Medicine and Caring Sciences, Linköping University, Linköping, Sweden; 2 Department of Radiology in Linköping, and Department of Health, Medicine and Caring Sciences, Linköping University, Linköping, Sweden; 3 Center for Medical Image Science and Visualization (CMIV), Linköping University, Linköping, Sweden

**Keywords:** Digital mammography, Cost-effectiveness, Breast cancer, Artificial intelligence

## Abstract

**Objective:**

To evaluate the cost-effectiveness of AI-assisted digital mammography (AI-DM) compared to conventional biennial breast cancer digital mammography screening (cDM) with double reading of screening mammograms, and to investigate the change in cost-effectiveness based on four different sub-strategies of AI-DM.

**Materials and methods:**

A decision-analytic state-transition Markov model was used to analyse the decision of whether to use cDM or AI-DM in breast cancer screening. In this Markov model, one-year cycles were used, and the analysis was performed from a healthcare perspective with a lifetime horizon. In the model, we analysed 1000 hypothetical individuals attending mammography screenings assessed with AI-DM compared with 1000 hypothetical individuals assessed with cDM.

**Results:**

The total costs, including both screening-related costs and breast cancer-related costs, were €3,468,967 and €3,528,288 for AI-DM and cDM, respectively. AI-DM resulted in a cost saving of €59,320 compared to cDM. Per 1000 individuals, AI-DM gained 10.8 quality-adjusted life years (QALYs) compared to cDM. Gained QALYs at a lower cost means that the AI-DM screening strategy was dominant compared to cDM. Break-even occurred at the second screening at age 42 years.

**Conclusion:**

This analysis showed that AI-assisted mammography for biennial breast cancer screening in a Swedish population of women aged 40–74 years is a cost-saving strategy compared to a conventional strategy using double human screen reading. Further clinical studies are needed, as scenario analyses showed that other strategies, more dependent on AI, are also cost-saving.

**Key Points:**

***Question*** *To evaluate the cost-effectiveness of AI-DM in comparison to conventional biennial breast cDM screening*.

***Findings*** *AI-DM is cost-effective, and the break-even point occurred at the second screening at age 42 years*.

***Clinical relevance*** *The implementation of AI is clearly cost-effective as it reduces the total cost for the healthcare system and simultaneously results in a gain in QALYs*.

**Graphical Abstract:**

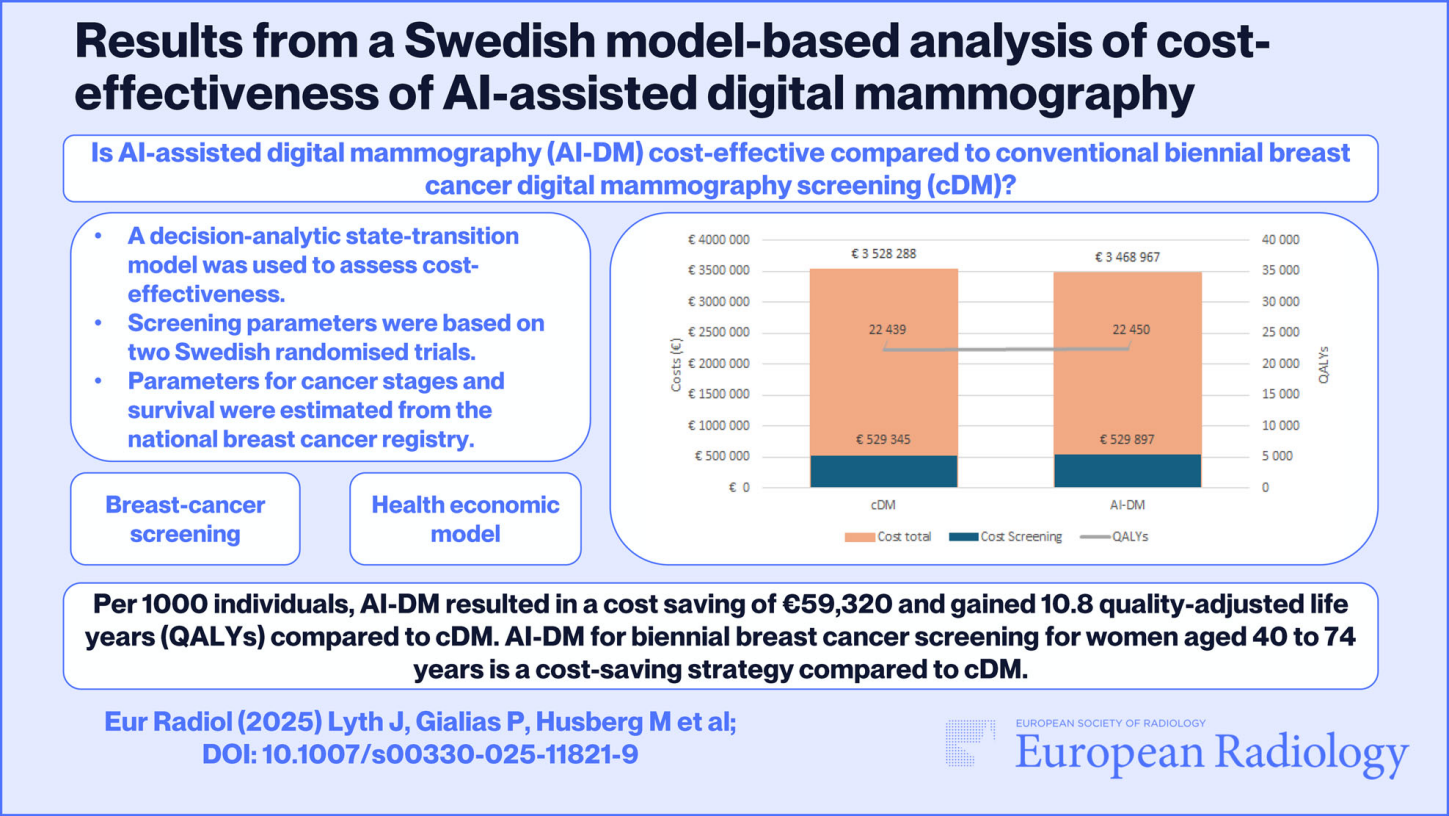

## Introduction

Worldwide, breast cancer accounts for almost one in four cancer cases among women [1].

Randomised clinical trials, systematic reviews, and observational studies have demonstrated that screening mammography reduces breast cancer-related mortality by 20–50% and has therefore been widely implemented [2–5]. The European guidelines recommend conventional biennial digital mammography (cDM) for women with double reading of mammograms [6]. All women registered in the Swedish healthcare system aged 40–74 years are invited to follow the national breast cancer screening programme [7]. The current double reading screening programme requires numerous radiologists [8, 9], but the global shortage of breast radiologists makes double reading difficult to sustain [10]. Additionally, up to 30% of cancers are not detected during screening and are eventually diagnosed as interval cancers between screening rounds [11, 12]. Assistance in screening interpretations is needed to reduce inter-observer variability, while also managing workforce limitations and maintaining diagnosis quality [13].

Due to the increasing need for workflow improvement in breast imaging, AI systems are rapidly being implemented in the clinical setting [14–21].

AI-based systems increase the effectiveness of breast cancer screening programmes [19]. In a recent Swedish randomised, controlled, population-based prospective clinical trial [15], the use of AI-assisted digital mammography (AI-DM) to triage screening examinations to single or double reading was regarded as non-inferior, as the cancer detection rate was above the prespecified lower safety limit, and the performance was similar to double reading cDM. Recall rates, false positives, and consensus meetings were not influenced, while the workload was reduced by almost half [15]. A recent systematic review and meta-analysis showed that standalone AI for screening digital mammography performed equal to or better than individual breast radiologists or average reader outcomes [22].

To estimate whether AI-DM is cost-effective, we need to consider cancer and screening-related costs using a lifelong perspective. As no long-term randomised trials exist comparing AI-DM vs cDM with two radiologists, a simulation model estimating long-term effects is required. To evaluate the value of AI technology in healthcare decision-making, a health economic analysis of AI-DM screening can be conducted by integrating clinical evidence, cost data, and health outcomes into a model.

The aim of this study is to evaluate the cost-effectiveness of AI-DM vs double reading cDM of mammograms using Swedish settings with biennial screening in the age interval of 40–74 years. We also investigated changes in cost-effectiveness based on four different sub-strategies of AI-DM.

## Materials and methods

In our analysis, the AI technology used for mammography screening is the deep learning algorithm Transpara (Version 1.7.0; ScreenPoint Medical). This commercially available AI system is Conformité Européenne (CE) marked, cleared by the U.S. Food and Drug Administration (FDA), and has been validated in previous studies (e.g. [15–18]). The AI system provides a continuous score from 0 to 98 (raw score) and CAD marks for suspicious regional findings of calcifications and soft-tissue lesions in each view of the breast. Based on the highest exam-level raw score, Transpara assigns an overall exam score on a scale from 1 to 10 to each exam, indicating the risk for malignancy. A score between 1 and 7 indicates a low risk, 8 to 9 indicates an intermediate risk, and 10 indicates an elevated risk for cancer. Transpara is calibrated in a way that roughly 10% of exams fall into each exam score. The AI system uses convolutional neural networks to analyse mammograms and has been trained on mammograms from different screening populations and multiple vendors. Many previous studies (e.g. [15–18]) have shown that this system can effectively and safely reduce the screening workload by triaging the cases and replacing one of the two radiologists for low-score mammograms.

### Model overview

A decision-analytic state-transition Markov model was used to analyse the decision problem of whether to use cDM or AI-DM in breast cancer screening. In each cycle, a patient is always in one of a finite number of health states, and events are represented as transitions from one state to another, going into the next cycle. Probabilities for events/transitions are based on the best possible knowledge. In this Markov model, one-year cycles were used, and the analysis was performed from a healthcare perspective with a lifetime horizon. In the model we analysed 1000 hypothetical individuals who were attending mammography screening, assessed with AI-DM, compared with 1000 hypothetical individuals assessed with cDM. The model started at age 40 years, and the screening procedure was repeated in the model every second year until age 74 years according to the Swedish national screening programme for breast cancer. The model presumed 100% adherence to screening, meaning that women who have undergone their initial screening continued to participate in all subsequent screenings. Reparticipation in the screening programme in Sweden exceeds 95%.

**Fig. 1:**
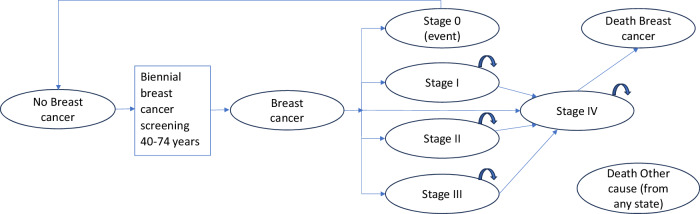
The Markov model is used. Circles represent specific health states in the model

Figure 1 shows the Markov model and health states. The model generates the following health economic outputs: incremental quality-adjusted life years (QALYs), estimation of the impact of AI-DM on patients’ quality of life (QOL) and life expectancy compared with cDM; incremental costs, the difference in costs for AI-DM compared with cDM; incremental cost-effectiveness ratio (ICER), calculation of the ICER, which represents the additional cost per unit of health outcome gained by implementing AI-DM screening compared to the traditional cDM approach. A probabilistic sensitivity analysis, using Monte Carlo simulation, was conducted by sampling all input parameters from their respective statistical distributions to simulate parallel cohorts of 1000 individuals. This approach integrates parameter uncertainty into the model, allowing for a comprehensive examination of the variability and robustness of the cost-effectiveness analysis results. In this health economic study, we followed the Consolidated Health Economic Evaluation Reporting Standards (CHEERS) 2022.

### Databases

The Swedish National Breast Cancer Register (NKBC) is a population-based registry that prospectively collects data on breast cancer diagnoses in Sweden. The registry includes detailed clinical information both on the primary cancer and on follow-up. Nearly all new breast cancer diagnoses in Sweden are included in the registry, with 98.5% registered within one year [23]. We have used the NKBC for many important breast cancer-related parameters in the breast cancer screening model. The Human Mortality Database (HMD) is the world’s leading scientific database on mortality and offers harmonised population and mortality estimates for over 40 countries.

### Screen reading, conferences, and recalls

Linköping University Hospital implemented the Transpara algorithm in 2021 and has decided to use double screen reading for intermediate and elevated risk for cancer (Transpara score 8–10). Therefore, we used 30% double human screen reading as the base-case value in our model, see Table 1. The proportion of recalls after cDM for individuals aged 40–54 years and 55–74 years was taken from a national Swedish mammography screening report [7]. A Swedish screening study showed no significant difference in recall rates between cDM and AI-DM, so we applied these proportions also for AI-DM [15]. We applied the same proportion of conferences for all ages, and the proportion of conferences after cDM and AI-DM was taken from a Swedish screening study [15].

**Table 1:** Screening-related parameters

	AI-DM	cDM	Assumed statistical distribution	Source
Screening-related proportions				
Proportion of 2 human readers	30%	100%	Fixed	Linköping University Hospital
Proportion of conferences	4.0%	3.9%	Beta (AI-DM: α = 1 475; β = 35 403 and cDM: α = 1 404; β = 34 590)	Lång et al 2023 [15]
Proportion of recalls 40–54 years AI	3.5%		Beta (α = 4 541; β = 125 208)	Screening Report [7], www.socialstyrelsen.se
Proportion of recalls 55–74 years AI	2.5%		Beta (α = 2 341; β = 91 297)	Screening Report [7], www.socialstyrelsen.se
Cancer detection risk				
Screening 40–74 years^#^	0.61%	0.51%	Beta (AI-DM: α = 222; β = 36 169 and cDM: α = 203; β = 39 576)	Lång et al 2023 [15]
Screening 40–54 years^#^	0.32%	0.27%	–	Lång et al 2023 [15]
Screening 55–74 years^#^	0.86%	0.72%	–	Lång et al 2023 [15]
Interval cancer risk 40–54 years*	0.03%	0.06%	–	Screening Report [7], www.socialstyrelsen.se
Interval cancer risk 55–74 years*	0.08%	0.15%	–	Screening Report [7], www.socialstyrelsen.se
Screening-related costs (€)				
Invitation cost	0.7		Fixed	Linköping University Hospital
Cost mammographic screening	30.5		Fixed	Linköping University Hospital
Human screen reading rate	29 per/h		Fixed	Linköping University Hospital
Cost for radiologist (per h)	79.0		Fixed	Linköping University Hospital
Cost of screen reading	2.7		Gamma (SD = 0.5)	Human screen reading rate/cost for a radiologist
Cost AI licence	1.9		Fixed	Contract, Linköping University Hospital
Cost conference	5.4		Gamma (SD = 1.1)	Linköping University Hospital
Cost recall	370.8		Fixed	Linköping University Hospital

^#^ Probability at biennial screening

* Annual probabilities

### Resource usage and unit costs

Table 1 lists all the unit costs used in the model. Costs related to the screening procedure included invitations to screening and mammography, including costs for staff, materials, and equipment. The screen reading cost was separated from the mammography cost as this was highly dependent on the number of screen readers. All unit costs related to screening were estimated by the economist affiliated with the radiology department at Linköping University Hospital. The hourly cost for a radiologist was estimated to be €79.0, and each radiologist was estimated to manage on average 29 individuals per hour, resulting in a screen reading unit cost of €2.7 per individual. The AI licence cost, excluding temporary discounts, was €1.9 million for Linköping University Hospital in 2023. Table 2 includes stage-specific breast cancer costs taken from a Swedish study [24]. We applied the same cost for stages 0, I, II, and III in the first year and the same cost for stages I, II and III in subsequent years. We were unable to find any data on the long-term cost for stage IV, so we applied the same cost for all years. All unit costs were adjusted to the year 2023 using the price index with quality-adjusted salaries for Swedish regional healthcare (LPIK) and converted to EUR using the mean exchange rate for 2023 (1 EUR = 11.5 SEK). A 3% discount rate was used for both costs and effects.

**Table 2:** Cancer-specific parameters

Distribution of stage for screening-detected cancer and interval cancer	Age	Screening	Interval	Age	Screening	Interval	Age	Screening	Interval
Stage 0	40–54	22.0%	7.3%	55–74	14.3%	6.5%	75+	–	4.7%
Stage I		48.0%	39.3%		62.4%	40.8%		–	34.1%
Stage II		21.0%	30.1%		17.3%	29.5%		–	38.4%
Stage III		8.6%	19.4%		5.6%	16.7%		–	16.3%
Stage IV		0.4%	4.0%		0.5%	6.5%		–	6.5%
	**Year**								
	1	2	3	4	5	6	7	8	9
Risk for distant metastasis									
Stage I	0.1%	0.4%	0.4%	0.4%	0.5%	0.5%	0.5%	0.5%	0.5%
Stage II	1.0%	1.8%	1.9%	1.9%	1.6%	1.6%	1.6%	1.6%	1.6%
Stage III	2.5%	7.6%	7.1%	5.9%	3.3%	2.8%	2.1%	2.0%	1.9%
Breast cancer-related death risk									
Stage IV	36.8%	32.4%	30.7%	33.9%	24.9%	6.8%	3.9%	3.9%	3.9%
Breast cancer cost ($)									
Stage 0	12,652								
Stage I, II, III	12,652	3489	3489	3489	3489	3489	3489	3489	3489
Stage IV	22,752	22,752	22,752	22,752	22,752	22,752	22,752	22,752	22,752
QOL decrement									
Stage 0	0.018								
Stage I, II	0.018	0.018	0.018	0.018	0.018	0.018	0.018	0.018	0.018
Stage III	0.077	0.077	0.077	0.077	0.077	0.077	0.077	0.077	0.077
Stage IV	0.099	0.099	0.099	0.099	0.099	0.099	0.099	0.099	0.099

Beta distributions were used for risk of distant metastasis, breast cancer-related death risk and QOL decrements. Gamma distributions were used for breast cancer cost. Data from the Swedish NKBC were used for years 1–5 for metastasis risk and death risk, and for years 6–9, we used data from Lord et al [27]

### Cancer detection risk

We base the cancer detection risk for AI-DM and cDM on a Swedish screening study [15]. However, this paper did not present any age-specific results, so we had to make some adjustments to obtain detection risk for ages 40–54 years and 55–74 years. From a Swedish screening report, we found that ages 40–54 years had 47% lower detection and ages 55–74 years had 40% higher detection than the average screening detection risk [7]. From the same report, we found that interval cancers accounted for 29.5% of all breast cancers in ages 40–74 years within a two-year period. We assumed that this proportion was consistent across both age groups, allowing us to estimate the total number of cancers for women aged 40–54 years and 55–74 years. We assumed that no additional cancers were detected by AI, and that the risk of interval cancer was calculated by subtracting the number of screening-detected cancers from the total number of cancers for both AI-DM and cDM, see Table 1. For individuals aged 75 years or older and no longer participating in the screening programme, we used age-specific breast cancer incidence data from the NKBC for the years 2018–2022. We used age-specific data for ages 75–90 years and applied the average incidence rate for individuals over age 90 years due to high uncertainty in the data for individual years. The stage distribution of newly detected, screening-detected, and interval cancer can be found in Table 2. Data on stage distribution were obtained from the NKBC, including 25,913 cases with detected breast cancer in different stages for the years 2020–2022.

### Utility weights

The age-specific standard QALY weights used in the model were attributed to the participants’ age based on a published study presenting the utility values of the overall population of Sweden [25]. Since the study by Burström et al [25] does not provide QALY weights for persons older than 88 years, we applied the same QALY weights for those aged 88 and above as for those at age 88. Table 2 presents QOL decrement per stage. To obtain these decrements, we used stage-specific QALY weights from a Swedish study with a mean age of 57 years and then subtracted this number from the standard QALY weight for a 57-year-old person [26]. We used the same decrement for stages 0, I, and II since stage-specific decrements could not be found in the literature.

### Transition probabilities, risk of distant metastasis for breast cancer patients

The NKBC registry contains a five-year follow-up on all breast cancer patients and, if the individual has a distant metastasis, the date of the first distant metastasis is recorded in the registry. We used this information and calculated stage-specific annual risk for distant metastasis using the Kaplan-Meier method for patients aged 40–74 years with primary breast cancer diagnosis in 2014–2017. The analysis was based on 11,459, 5916, and 433 patients in stages I, II, and III, respectively. We assume in the model that stage 0, by definition, cannot lead to a distant metastasis. We saw from an Australian study that the probabilities were stable after 5 years for stages I and II, so we used the same probability after 5 years [27]. For stage III, we had very similar probabilities as Lord et al [27], so we simply applied the Australian probabilities after five years.

### Death probabilities

Age-specific standard mortality rates were taken from the HMD using Swedish life tables for the year 2022 (mortality.org). We used data from the NKBC with 977 registered first distant metastases (stage IV) between 2014 and 2017 and estimated the annual breast cancer-specific death risks up to five years using the Kaplan-Meier method. Patients were followed up until 31 December 2022 and were censored if death was from a cause other than breast cancer. We limited the analysis to five years due to the few remaining cases that cause high uncertainty. Instead, we used data after 5 years from the Australian study (Lord et al [27]).

### One-way scenario analyses

In addition to the base-case analysis, we applied four different scenario analyses. In the first scenario, we assessed the changed costs associated with the use of double human screen reading only for elevated risk for cancer (Transpara score 10), as in Lång et al [15]. In the second scenario, we tested using only a single human screen reader plus AI for all individuals. A Swedish study showed that single human screen reading plus AI was associated with a 3% lower relative frequency (261/269) compared with using only double human screen reading plus AI [19]. In this analysis, we therefore lowered the cancer detection probability by 3% for AI-DM. In the third scenario, we tested using AI for risk 0–7 and single human screen reading, plus AI for risk 8–10. A Swedish study showed that a single human screen was associated with a 9% lower relative frequency (246/269) compared with using only a double human screen reading plus AI [19]. In this analysis, we therefore lowered the cancer detection probability by 9% for AI-DM. In the fourth scenario, we used the base case scenario but assessed the costs and effects of outsourcing all screen reading to an external healthcare company with a unit cost of €4.8.

## Results

### Base-case scenario

The total costs, including both screening-related costs and breast cancer-related costs, were €3,468,967 and €3,528,288 for AI-DM and cDM, respectively. AI-DM resulted in a cost saving of €59,320 compared to cDM. Per 1000 individuals, AI-DM gained 10.8 QALYs compared to cDM. Gained QALYs at a lower cost means that the AI-DM screening strategy was dominant compared to cDM. The break-even occurred at the second screening at age 42 years. Biennial screening of 1000 individuals between 40 and 74 years of age resulted in 11,810 fewer human screen readings for AI-DM compared with cDM, see Table 3. This corresponds to €20,703 in lower costs, but the licence cost for AI amounted to €20,840, which means that there was no total cost saving for screen readings. Overall, the AI-DM strategy was slightly more expensive than cDM, with an incremental cost of €552.

**Table 3:** Base-case results are deterministic (per 1000 screened)

Frequency		AI-DM	cDM	Incremental
Screening	No. of mammographs	16,970	16,953	17
	No. of AI screen readings	16,970	0	16,970
	No. of human screen readings	23,419	35,229	−11,810
	No. of conferences	679	661	18
	No. of recalls	503	503	0
Costs (€)				
Initial	Cost invitations	7137	7131	6
	Cost mammographs	333,062	332,801	261
	Cost human screen readings	41,027	61,729	−20,703
	Cost AI	20,840	0	20,840
	Cost conferences	2378	2317	61
Recall	Cost invitation	221	221	0
	Cost of other diagnostics	125,232	125,146	86
Breast cancer	Cost stage 0	133,505	121,858	11,647
	Cost stage I	1,486,650	1,428,447	58,203
	Cost stage II	625,247	662,549	−37,301
	Cost stage III	276,034	314,722	−38,687
	Cost stage IV	417,634	471,368	−53,734
Screening-related costs		529,897	529,345	552
Breast cancer-related costs		2,939,071	2,998,943	−59,872
Total cost (screening and breast cancer)		3,468,967	3,528,288	−59,320
QALYs		22,450	22,439	10.8
ICER				−5506 (dominant)

### Probabilistic sensitivity analysis

**Fig. 2:**
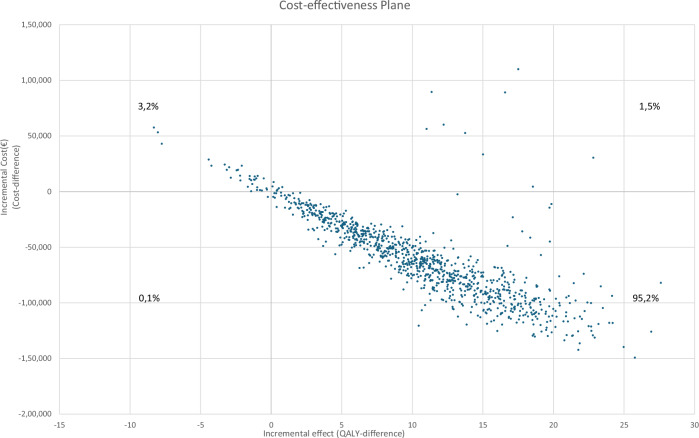
Incremental cost-effectiveness plane showing 1000 Monte Carlo estimates of incremental costs per individual and benefits of AI-assisted biennial breast cancer digital mammography screening for ages 40–74 years using two radiologists only for Transpara score 8–10 (AI-DM) compared to conventional biennial breast cancer digital mammography screening for ages 40–74 years using two radiologists (cDM). Screening with AI-DM was dominant in 95.2% of the simulations compared to cDM

Figure 2 shows the incremental cost-effectiveness plane from 1000 Monte Carlo estimates of incremental costs per individual and benefits for AI-DM compared to cDM. The result from the probabilistic sensitivity analysis showed that the AI-DM strategy was dominant in 95.2% of the simulations. This means that it is 95.2% certain that AI-DM is both less costly and produces more health (QALYs) for the individuals compared to cDM. AI-DM was less effective (fewer QALYs) in 3.3% of the simulations compared to cDM, and in 1.5% AI-DM was more effective but at a higher cost compared to cDM. The AI-DM strategy was cost-effective, if willingness to pay is set to €50,000/QALY, in 96.7% of the simulations.

### One-way scenario analyses

**Fig. 3:**
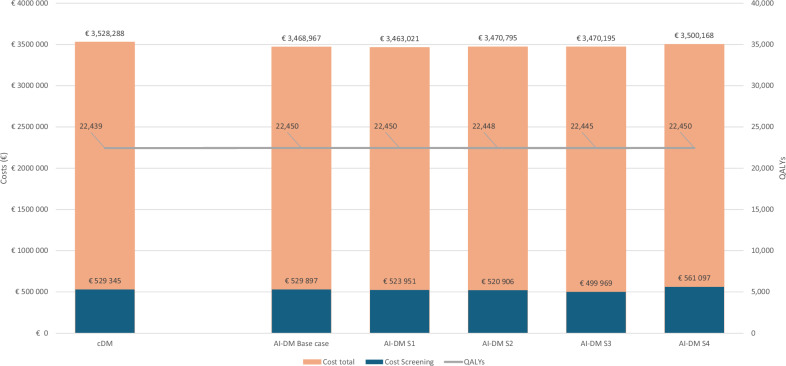
Costs and QALYs gained by conventional biennial breast cancer digital mammography screening for ages 40–74 years using two radiologists (cDM) compared with screen-reading procedure using AI-DM with double human screen reading only for Transpara score 8–10 (AI-DM) and four other scenarios (S1–S4). S1: AI-assisted mammography with double human screen reading only for Transpara score 10. S2: AI-assisted mammography with only a single reading independent of Transpara score. S3: AI-assisted mammography without human screen reading for Transpara score 0–7 and single human reading for Transpara score 8–10. S4: base-case scenario but with outsourcing of all human screen reading to an external company with a unit cost of €4.8

The base-case scenario included double human screen reading for both intermediate and elevated risk for cancer (Transpara score 8–10). In scenario analysis one, increasing the threshold level to only use double human screen readers for elevated risk for cancer (Transpara score 10) resulted in a cost saving related to screening of €5394. Scenario analysis two, where we only used single human screen reading plus AI for all individuals, resulted in a screening-related cost saving of €8439. However, in the second scenario, the number of gained QALYs was reduced from 10.8 to 8.8 compared to the base-case scenario. Scenario analysis three, where we used only AI for risk 0–7 and used one human screen reading for risk 8–10, resulted in the largest screening-related cost saving, €29,376. The number of gained QALYs was thus reduced from 10.8 to 5.2. In the fourth scenario analysis, we applied the base-case scenario but outsourced the screen reading to another company with a consultant fee of €4.8 per examination. The strategy with AI-DM then resulted in an increased screening-related cost of €31,752 but gave cost savings of €28,120 if also cancer costs were considered. The results of the four scenarios can be found in Fig. 3.

## Discussion

A Markov model was used to simulate lifetime costs and effects of inviting 1000 women to biennial screening using either cDM or AI-DM. The analysis showed that AI-DM was a dominant strategy, i.e. gaining QALYs and saving costs compared to cDM. The analysis revealed that while screening-related costs were marginally higher, the transition to earlier cancer detection resulted in reduced long-term costs within the model.

Although the use of AI in mammography is an emerging field and several randomised screening trials have been published, we have only identified one previously published article that evaluates the long-term cost-effectiveness of AI-assisted mammography [28]. The British study evaluated the cost-effectiveness of the machine-learning system called mammography intelligent assessment from Kheiron Medical Technologies, replacing one radiologist with AI, compared to double human screening reading within the NHS system. They found that the AI system was cost-effective with lower costs but also with lower QALYs. However, the model used was highly conservative, resulting in fewer cancer detections by the AI compared to the standard NHS strategy. Two large, randomised screening trials from Sweden have recently shown that AI systems (Transpara and Lunit) in combination with a radiologist detect more cancer than cDM with double human screen reading [15, 19]. One frequent hypothesis is that AI systems find cancers that otherwise would have been found as interval cancers. Lång et al [17] reviewed 429 interval cancers and found a statistically significant correlation between the interval cancer classification (minimal sign/false negative) and the AI risk score. The AI system correctly located 58% of these cases and found 23% of the stage IV cancers and/or women who died from their cancer. This suggests that AI could potentially reduce the interval cancer rate and improve the detection of high-risk disease. AI systems can possibly contribute to minimising overdiagnosis, which is one of the major limitations of mammography screening (4), and positively affect the cost-effectiveness and QALY results for the AI-DM. More studies are needed to confirm these hypotheses.

If we assume that AI-DM does not detect more cancer than cDM, the shortage of radiologists might justify the marginally higher cost of AI-DM. The major strength of this study is that our simulation model was based on two Swedish randomised trials, which gives reliable estimates of cancer detection rates with AI [15, 19]. Further, parameters of cancer incidence, cancer-related death, and the distribution of stages from screening and spontaneous detection were estimated from high-quality population-based Swedish reports and registries. There have been significant advances in the treatment of stage IV breast cancer in recent years. However, contemporary cost data for this parameter are lacking, likely resulting in an underestimation of the costs associated with disseminated disease. However, if the AI system detects cancer in earlier stages, this underestimation could be considered conservative. Another limitation is that cost data were highly dependent on the Swedish healthcare system, potentially limiting the generalisability of these findings to other healthcare systems.

The results show that the implementation of AI is similar to cDM in terms of screening costs, total costs, and QALYs regardless of the strategy for implementing AI (Fig. 3). However, even though the differences are small, the implementation of AI is clearly cost effective as it reduces the total cost for the healthcare system while simultaneously resulting in a gain in QALYs for the base case and scenarios 1–4 (Figs. 2 and 3).

An important finding is that scenario 2 (AI-assisted mammography with only single reading independent of Transpara score) performed worse than the base case and scenario 1 in terms of total costs and QALYs, despite marginally lower screening costs. This outcome is particularly noteworthy as some AI-DM vendors advocate for this type of implementation.

An important limitation of this study pertains to the estimation of screen-reading unit costs per individual. The estimation is derived from the hourly cost of a radiologist and the projected number of readings conducted per hour. Both projections are based on the average for all screen readers in the Östergötland region of Sweden. The number of screen readings per hour utilised in this study is consistent with figures described in a British study [28]. However, the radiologist's cost per hour is higher in Britain, rendering the cost per screen reading comparable to the cost for external reading in Sweden. It is worth noting that other activities in which screen readers are involved, such as clinical work, the training of resident doctors, and administrative duties, have not been accounted for in this analysis.

Although the present study is situated within the Swedish healthcare system, it is important to consider the potential implications and generalisability of the findings to other healthcare contexts, particularly in relation to differing reimbursement structures and variations in radiologist workforce capacity. Sweden operates a publicly funded healthcare system characterised by a high degree of centralisation, with standardised clinical pathways and a strong emphasis on equitable access to care. Such a framework facilitates the uniform adoption of diagnostic strategies and supports interventions aimed at optimising resource utilisation.

In contrast, healthcare systems in other countries—particularly those with mixed or predominantly private funding models, such as the United States—may face distinct challenges in translating these findings into practice. In such systems, reimbursement mechanisms and financial incentives can substantially influence diagnostic decision-making, potentially prioritising volume over value-based care. As a result, interventions that are cost-effective within a publicly funded model may encounter barriers to implementation where reimbursement is procedure-based rather than outcome-driven.

Moreover, the availability of radiologists constitutes a critical variable in assessing the transferability of these findings. Sweden, like many European countries, is experiencing a shortage of radiologists, prompting the need for strategies that enhance diagnostic efficiency and optimise the use of available expertise. However, the magnitude of radiologist shortages varies internationally, and healthcare systems with a higher radiologist-to-population ratio may experience different pressures and priorities. Conversely, in settings where workforce constraints are even more acute, the relevance and urgency of workflow optimisation and task prioritisation could be even greater.

Additional factors influencing generalisability include the degree of digitalisation of healthcare systems, the integration of clinical decision support tools, medico-legal frameworks, and cultural attitudes toward diagnostic uncertainty and clinical risk. These contextual elements must be carefully considered when extrapolating findings beyond the Swedish system.

While the results of this study are highly pertinent within the Swedish context, extrapolation to other healthcare systems should account for structural, economic, and workforce-related differences. Future research examining the adaptation and effectiveness of similar strategies in diverse healthcare environments would further inform their broader applicability.

Future research efforts should prioritise prospective, multicentre studies to validate the clinical and economic impact of AI applications. This approach will inform evidence-based updates to European screening policies and facilitate equitable access to high-quality breast imaging services.

The potential of artificial intelligence in digital mammography will be fully realised only through careful policy adaptation, rigorous clinical validation, and a sustained commitment to delivering equitable, patient-centred care.

## Conclusion

This analysis showed that the AI-assisted mammography for biennial breast cancer screening in a Swedish population of women aged 40–74 years is a cost-saving strategy compared to a conventional strategy using double human screen reading. Further clinical studies are needed, as scenario analyses showed that other strategies, more dependent on AI, are also cost-saving.

## Abbreviations

AI-DM: AI-assisted digital mammography
HMD: Human mortality database
ICER: Incremental cost-effectiveness ratio
NKBC: National Breast Cancer Register
QOL: Quality of life
QALYs: Quality-adjusted life years
